# Development and Validation of a GC-EI-MS/MS Method for Ethyl Glucuronide Quantification in Human Hair

**DOI:** 10.3389/fchem.2022.858205

**Published:** 2022-04-04

**Authors:** Alessandro Mattia, Clementina Moschella, Maria Chiara David, Marco Fiore, Sara Gariglio, Alberto Salomone, Marco Vincenti

**Affiliations:** ^1^ Dipartimento della Pubblica Sicurezza, Direzione Centrale di Sanità, Centro di Ricerche e Laboratorio di Tossicologia Forense, Ministero dell’Interno, Roma, Italy; ^2^ Institute of Biochemistry and Cell Biology, IBBC—CNR, Rome, Italy; ^3^ Dipartimento di Chimica, Università Degli Studi di Torino, Torino, Italy

**Keywords:** EtG, hair, GCMS/MS, ethyl glucuronide, alcohol consumption, drinking habits

## Abstract

Ethyl glucuronide (EtG) is a minor, non-oxidative ethanol metabolite detectable in several matrices for specific periods of time. In recent years, quantification of EtG in hair has been established as the most reliable biomarker for long-term alcohol consumption, with the Society of Hair Testing offering cut-off values for assessment of both abstinence and heavy drinking. Instrumental constrains and wide inter- and intra-laboratory variability represent the ultimate barriers to widespread acceptance of hair EtG determination in the forensic context. In this study, a new analytical method for hair EtG based on gas chromatographic (GC) separation, electron impact (EI) ionization, and tandem mass spectrometry (MS/MS) detection was developed and validated. At the same time, several parameters for sample pretreatment and instrumental analysis were optimized using real hair samples obtained from different drinking subjects. A full-factorial design-of-experiment approach included procedures for hair washing, pulverization, and extraction. Rigorous multi-step washing proved not to reduce the EtG content extracted in the subsequent sample incubation. Hair pulverization with a ball mill significantly improved the EtG extraction from the keratin matrix and allowed us to reduce the time needed for the subsequent extraction step, without affecting the extraction recovery. The hair extract was derivatized with N-methyl-N-(trimethylsilyl)-trifluoroacetamide. Upon electron impact ionization of the EtG-TMS derivative, triple quadrupole mass analyzers were operated in the selected reaction monitoring (SRM) mode using the fragment m/z 405 as the precursor ion (m/z 410 for the EtG-D5 internal standard), the transitions m/z 405 → 359 and m/z 410 → 359 for quantitation, and m/z 405 → 331 and m/z 405 → 287 for qualification/confirmation, all at 10 V collision energy. The final method was fully validated and then applied to 25 real hair samples. The calibration curve proved linear between 6 and 60 pg/mg. The limit of detection (LOD) was 4 pg/mg. Intra- and inter-assay precision and accuracy tests showed a variability and bias close to 15% or lower over the entire calibration range. The new method is routinely applied in the Italian State Police’s toxicology laboratory for hair analyses addressed to exclude excessive alcohol drinking and verify the psycho-physical requirements of the personnel.

## Introduction

Alcohol abuse is a source of major concern in most countries because it produces a multitude of serious accidents, crimes, and pathologies. In 2018, the World Health Organization stated that three million worldwide deaths (5.3% of the total) were linked to alcohol abuse, with mortality particularly related to hazardous drinking and a significant increase (up to 13.5%) among young people (WHO, 2018). In this perspective, there is an increasing need for police forces and toxicology laboratories to detect the condition of alcohol abuse by means of sensitive and specific biomarkers (Cabarcos et al., 2015; Wurst et al., 2015; Andresen-Streichert et al., 2018; Mastrovito and Strathmann, 2020) and effective analytical methods, affordable by variably equipped laboratories (Vincenti et al., 2013; Alladio et al., 2017).

Ethyl glucuronide (EtG) is a minor non-oxidative ethanol metabolite (≤1% of ingested ethanol) (Woźniak et al., 2019; Fosen et al., 2016), resulting from enzymatic glucuronidation of ethanol in the liver (Woźniak et al., 2019). This small, polar, slightly acidic, and relatively stable molecule can be detected in several body fluids and tissues for variable time intervals after ethanol ingestion: blood and urine EtG levels are commonly used to assess short-term consumption (up to a few days), whereas the keratinized matrices (mainly hair) are increasingly employed to monitor abstinence and chronic abuse (over months) (Nanau and Neuman, 2015). Reliable quantification of EtG in both traditional (i.e., blood and urine) and non-traditional (i.e., hair and nails) matrices is typically carried out using gas chromatographic (GC) and/or liquid chromatographic (LC) separation coupled to single or tandem mass spectrometry (MS) (Nanau and Neuman, 2015; Bager et al., 2017). Since its introduction, hair EtG has become a common marker of alcohol consumption in both clinical and forensic settings (Kharbouche et al., 2011; Morini et al., 2011; Crunelle et al., 2014; Society of Hair Testing, 2016; Bager et al., 2017; Verbeek et al., 2018; Woźniak et al., 2019). A growing interest is observed also in epidemiological studies that investigate the correlation between drinking patterns and chronic disease and mortality (Crunelle et al., 2014). Currently, the consensus of the Society of Hair Testing (Society of Hair Testing, 2016) reports that EtG concentration in hair is expected to exceed a cut-off value of 30 pg/mg for chronic excessive drinkers, approximately equivalent to an average consumption of 60 g or more of pure ethanol per day over several months.

The aim of this study was to develop and validate a sensitive and specific analytical method based on gas chromatography coupled with electron impact–tandem mass spectrometry (GC-EI-MS/MS) for the determination of EtG in hair as a quantitative biomarker of alcohol consumption.

After an extensive analysis of previous literature (Table 1), twelve published methods for EtG determination on the keratin matrix based on GC separation were selected for comparison with the present one. Among these, six used a triple quadrupole (QqQ) mass analyzer. The ionization was carried out either by electron-capture negative chemical ionization (NCI, 8 methods) or EI (4 methods). Among the latter, only two presented a GC-EI-MS/MS configuration similar to the one adopted in the present study, but used a different reagent for EtG derivatization and different MS/MS transitions. In general, widely different operating conditions were used in the methods selected for comparison, including those utilized for preliminary hair sample treatments, EtG extraction, and derivatization. A critical revision of these operating conditions was conducted within the present study to achieve optimal performance features.

**TABLE 1:** Overview of the available methods to carry out EtG quantification in GC.

References	Type of sample	Amount of sample	Decontamination	Homogenization	Extraction	Clean-up	Derivatization	Analytical technique	Ions/transitions
Vignali et al. (2018)	Hair	30 mg	1 ml dichloromethane, 1 ml methanol	Milling	1 ml deionized water (2 h sonication)	Strata X-A SPE	75 μl PFPA, 50°C, 60’	GC-NCI-MS/MS	Quant 347→163–352→163 (d_5_), qual 347→119
Cappelle et al. (2015)	Hair	30 mg	Deionized water, acetone	Milling	2 ml deionized water (1.5 h sonication)	Oasis MAX SPE	HFBA, 60°, 30’	GC-NCI-MS/MS	Quant 596→213–601→213 (d_5_), qual 397→213
Mönch et al. (2013a)	Hair	50 mg	1 ml dichloromethane, 1 ml methanol (after cutting)	Cutting	0.5 ml deionized water (2 days, room temperature)	Lyophilization	100 µl PFPA, 60°C, 45’	GC-NCI-MS	Quant 496–501 (d_5_), qual 347
Paul et al. (2010)	Hair	20 mg	Methanol	Cutting	1 ml deionized water (overnight sonication)	Oasis MAX SPE	10 µl BSTFA + 10 µl ethyl acetate, 80°C, 20’	GC-EI-MS/MS	Quant 261→143–266→143 (d_5_)
Agius et al. (2010)	Hair	10–50 mg	2 ml deionized water, 2 ml acetone	Milling	2 ml water (2h sonication, 40°)	Clean Screen EtG SPE	40 µl HFBA, 80°C, 15’	HS-SPME-GC-NCI-MS/MS	Quant 596→427–601→432 (d_5_), qual 596→288–601→288 (d_5_)
Kharbouche et al. (2009)	Hair	30 mg	Deionized water, acetone	Milling	1 ml deionized water (2 h sonication)	Oasis MAX SPE	100 µl PFPA, 80°C, 30’	GC-NCI-MS/MS	Quant 347→163–352→163 (d_5_), qual 347→119
Álvarez et al. (2008)	Hair	100 mg	5 ml 0.1% polysorbate	Cutting	4 ml deionized water, 4 ml hexane (microwave-assisted extraction, 110°C, 11’)	—	BSTFA	GC-EI-MS	Quant 261–266 (d_5_), qual 160, 405
			80 (2x), 5 ml deionized water (2x)						
Paul et al. (2008)	Hair	10 mg	Methanol	Cutting	1 ml deionized water (overnight sonication)	Waters Oasis	10 μl BSTFA + 10 μl ethyl acetate, 80 °C, 20’	GC-EI-MS/MS	Quant 261→134–266→143 (d_5_)
						MAX SPE			
Kerekes et al. (2008) Martins Ferreira et al. (2012)	Hair	30 mg	Deionized water, acetone	Milling	2 ml deionized water (2 h sonication)	Oasis MAX SPE	PFPA, 60°C, 30’	GC-NCI-MS	Quant 496–501 (d_5_), qual 349
Jurado et al. (2004)	Hair	100 mg	Deionized water, acetone	Cutting	2 ml deionized water (2 h sonication, overnight incubation, room temperature)	—	100 μl of PFPA, room	GC-EI-MS	Quant 333–338 (d_5_), qual 234, 495
							temperature, 30’		
Yegles et al. (2004)	Hair	30 mg	Deionized water,	Milling	2 ml deionized water (2 h sonication)	Isolute NH2 SPE	100 μl PFPA + PFPOH 70 μl, 90°C, 30’	GC-NCI-MS	Quant 496–501 (d_5_), qual 347
			heptane						
Mönch et al. (2013b)	Hair	50 mg	1 ml dichloromethane	Milling	0.5 ml deionized water (48 h	—	PFPA	GC-NCI-MS	Quant 496–501 (d_5_), qual 347
			1 ml methanol		incubation, room temperature)				

PFPA = pentafluoropropionic anhydride, HFBA = heptafluorobutyric anhydride, BSTFA = N,O-bis(trimethylsilyl)trifluoroacetamide.

## Materials and Methods

### Materials

EtG, and its penta-deuterated analog EtG-D5, used as the internal standard (100 mg/L solutions in methanol), were obtained from Medichem (GmbH & Co., Steinenbronn). EtG and EtG-D5 working solutions were prepared by dilution in methanol at a concentration of 100 pg/μL and stored at −20°C. The stability of the analytes was checked before the analysis of each new batch of sample.

Solid phase extraction polymeric cartridges Strata X-A-33 µm were obtained from Phenomenex Inc. (Torrance, California). Methanol, dichloromethane, ammonia (28%), formic acid, and water for extraction were analysis grade pure and obtained were from Sigma-Aldrich (St. Louis, Missouri). N-Methyl-N-(trimethylsilyl)-trifluoroacetamide (MSTFA) used as the derivatizing agent was also obtained from Sigma-Aldrich.

### Hair Sample Decontamination and Extraction Procedure

Hair samples were decontaminated using two different approaches:

1) Two 10-min washes with 5 ml dichloromethane followed by two 10-min washes with 5 ml methanol, each performed in an inert glass tube, stirred on a rotator at 15–20 rpm;
2) One 1-min wash in 5 ml dichloromethane performed in an inert glass tube with a vortex mixer.

Then, the hair samples were air-dried. The effect of decontamination and possible washout of the analyte from the matrix were investigated by varying the final optimized conditions.

Washed hair samples (50 mg) were shredded using two alternative methods:

1) Fine cutting (1–2 mm length) with scissors;
2) Rough cutting with scissors followed by pulverization in a ball mill for 10 min.

The resulting cut or pulverized sample was transferred to a glass tube for the extraction procedure, following two alternative protocols:

1) Extraction overnight at 60°C with 2 ml of deionized water;
2) Extraction for 2 h under sonication at 50°C with 2 ml of deionized water.

Regardless of the procedure chosen to treat the sample, 15 µl of EtG-D5 working solution was added before the extraction. The liquid extract was recovered upon centrifugation at 4500 rpm for 10 min.

The performance and potential application of the aforementioned alternative procedures were studied following a repeated full-factorial experimental design (DoE) for three sets of homogeneous samples obtained from three different donors, previously tested for EtG. The k = 3 experimental parameters studied included 1) washing procedure, 2) shredding method, and 3) incubation and extraction conditions. The summary of the parameters’ setting is reported in Table 2. Each variable setting was indicated with the symbols “high” (+) or “low” (−).

**TABLE 2:** Values of the variables considered for the optimization of the pretreatment protocol.

Parameter	HIGH (+)	Low (−)
(a) Washing	2 × 5 ml CH_2_Cl_2_ followed by 2 × 5 ml CH_3_OH washes for 10 min each in inert glass tubes on an automatic rotator at 15–20 rpm	1 × wash in 5 ml CH_2_Cl_2_ rapidly vortexed for 1 min in inert glass tubes
(b) Incubation	Overnight at 60°C	2 h Sonication at 50°
(c) Shredding	Roughly cut and pulverized in a ball mill for 10 min	Cut with a pair of scissors at 1–2 mm

Each variable refers to a step; in particular, “a” represents the washing method, “b” the incubation, and “c” the sample shredding technique. For each variable, two levels called low (−) and high (+) were considered, respectively, relating to a mild or strong treatment.

### Extract Purification and Derivatization

Purification and concentration of EtG were carried out with SPE cartridges conditioned with 2 ml of water and 2 ml of methanol before sample loading. Special care was taken to avoid column drying between the conditioning and loading steps. The SPE cartridge was then washed with 2 ml NH_3_ 0.1 mM. A vacuum of −0.5 bar was applied for 5 min to remove all residual water, and another washing step with 1 ml of methanol was performed under vacuum to remove the last traces of humidity. Elution was performed with 2 ml of a formic acid/methanol solution (1:99 v/v). The eluate was then evaporated to dryness under a gentle stream of nitrogen at 40°C for 15 min. The residue was dissolved with 20 μL of acetonitrile, and 30 μL of MSTFA was added to a 4-ml closed-cap vial. The derivatization step was carried out at 80° for 40 min, and 2 µL of the cooled final solution was injected into the GC-EI-MS/MS system.

### GC-EI-MS/MS

An Agilent Technologies 7890B gas chromatograph coupled to a 7000C tandem mass selective detector operating in EI ionization mode was used for the analyses. The GC separation was achieved by a Phenomenex Zebron 30 m × 250 µm × 0.25-µm column with a (5%-phenyl)-methylpolysiloxane stationary phase. The injection temperature was set to 220°C and the injection volume was 2 µL. A pulsed-splitless injection was used at 20 psi for 0.75 min. The solvent delay was about 3 mins. The oven temperature was programmed as follows: isothermal at 100°C for 1 min, then ramped at 30°C/min up to 200°C, held for 0 min, ramped again at 15°C/min to 290°C, and final isothermal at 290°C for 3 min. The total chromatographic run adds up to 13.3 min. The transfer line was held at 280°C and the EI source at 230°C.

### Calibration and Validation Procedures

The procedure adopted for the analytical method validation complied with both “*The Fitness for Purpose of Analytical Methods*” (Magnusson and Örnemark, 2014) and “*The International Recommendations for Validation of New Analytical Methods*” (UNODC, 2009). Calibration data-points were obtained by analyzing extracted blank hair samples spiked with appropriate amounts of EtG working solutions and 15 µl of EtG-D5 working solution to produce five concentration levels at 6, 10, 20, 30, and 60 pg/mg. The homoscedastic distribution of data-points was assessed from Fischer’s test results on the calibration data. Therefore, no weighting factor was adopted for the linear regression model. The linearity of the regression model was verified by lack-of-fit and Mandel’s tests at a significance level of α = 0.05.

Intra- and inter-assay precision and trueness were assessed at medium and high concentrations (20, 30, and 60 pg/mg). Intra-day parameters were calculated by analyzing three concentrations in three replicates. Inter-day precision and trueness were estimated by analyzing the same three concentrations on 3 different days, operated by three different operators. Relative standard deviation (RSD) and percentage deviation of the average concentration from the corresponding nominal value were used to estimate precision and accuracy, respectively.

The detection limit (LOD) was defined as the lowest concentration giving a signal at least three times higher than the average of the baseline noise (S/N > 3, as determined by MassHunter software with the Auto-RMS algorithm). The LOD was also calculated using the Hubaux–Vos algorithm. Both approaches gave a comparable result. The limit of quantification (LOQ) was defined as the lowest concentration that could be measured with an intra-assay precision CV% and a relative bias less than 20%; in practice, the LOQ corresponded to the lowest level of the calibration curve. Hair samples from teetotalers were analyzed to detect possible interfering peaks in the signal. Both untreated and dyed hairs were tested for detecting potential interferences.

### Population and Sampling

Ethical approval for the study was granted by the Ethical Committee of the University of La Sapienza, Rome, Italy. All donor subjects (*N* = 25) gave their informed consent to take part in this study and completed a survey on their drinking habits. All samples and donors were anonymized before the analysis.

Volunteers were both 1) addiction treatment service patients from the Hospital Alcohol Center “Umberto I,” Rome, and 2) laboratory personnel (either teetotalers or with moderate drinking habits). Following the work of Paul et al. (2010), the amount of alcohol that the volunteers drank on a regular basis was reported in approximate units (1 alcoholic unit = 12 g of pure alcohol) per week. On the basis of the donors’ declaration, they were classified into five categories:

Heavy drinker: >25 units per week—three subjects.

Moderate drinker: >15 units per week—six subjects.

Social drinker: >10 units per week—four subjects.

Light drinker: >5 units per week—six subjects.

Teetotaler: 0 units per week—six subjects.

A strand of hair with a diameter of 3–4 mm was tied by a string and carefully cut with scissors, directly at the skin surface, preferably at the vertex posterior, in accordance with international recommendations (Cooper et al., 2012). All samples were stored under dry and dark conditions at room temperature until analysis. The segment up to 6 cm was analyzed.

## Results and Discussion

### Comparison of GC-MS Analytical Methods

Table 1 presents twelve analytical methods extracted from the published literature that use GC separation for EtG determination in hair. These methods are used for comparison with the present procedure.

In the twelve methods under comparison, the mean amount of hair used for the analysis was 42.5 mg, varying from 10 to 100 mg. The hair decontamination procedures were diverse and involved different solvents and steps since a globally accepted procedure does not exist yet (Kintz et al., 2015). A large combination of solvents, volumes, and decontamination times were proposed. Five procedures involved homogenization of the hair sample by manual cutting into short segments (Shi et al., 2010; Kronstrand et al., 2012), while seven methods used a ball mill to pulverize it (Mönch et al., 2013a; Mönch et al., 2013b; Agius et al., 2010) so as to maximize EtG recovery. EtG extraction from the keratin matrix was carried out by incubation with deionized water, except when microwave-assisted extraction was employed, but the timing and temperature of the extraction procedure varied significantly among the different methods. Also, different choices of the derivatizing agent was observed, including pentafluoropropionic anhydride (PFPA; 7/12, 58%), heptafluorobutyric anhydride (HFBA; 2/12, 17%), and N,O-bis(trimethylsilyl)trifluoroacetamide (BSTFA; 3/12, 25%), in different volumes, reaction times, and temperatures.

Extraction of efficient analytes from the inner layers of the hair matrix represents one of the most critical issues in hair analysis (Jurado et al., 2004) (Polettini et al., 1997), as is confirmed by several studies concerning the impact of sample preparation on quantitative EtG determination (Kronstrand et al., 2012; Salomone et al., 2016; Mueller et al., 2017; Woźniak et al., 2019). Several factors affect the extraction yield, including the hair sample size, shred method (powdering or snipping), duration, temperature, and incubation technique (Albermann et al., 2012; Alladio et al., 2018; Mönch et al., 2013c; Kummer et al., 2014; Becker et al., 2018). Occasionally, the published protocols do not report crucial details, leaving data interpretation unresolved (Müller and Iwersen-Bergmann, 2020) and resulting in considerable inter-laboratory variability during proficiency tests for hair EtG (Sporkert, 2011). Yet, an interlaboratory comparison, regardless of the analytical method, is possible (Becker et al., 2018).

### Derivatization Products and Selected Reaction Monitoring Detection

Most analytical methods for EtG are conducted via electrospray ionization (ESI) with LC-ESI-MS/MS instrumentation (Morini et al., 2006; Lamoureux et al., 2009; Albermann et al., 2010; Pirro et al., 2013; Imbert et al., 2013) or GC-NCI-MS/MS (Kerekes et al., 2008; Kharbouche et al., 2009; Martins Ferreira et al., 2012); precursor and product ions selected for both ionization techniques are not eligible in the EI mode.

The selected ion (reaction) monitoring used for quantitative and qualitative EtG detection clearly depended on the chosen ionization technique, derivatization reagent, and stages of MS analysis. Limiting the discussion to the two existing GC-EI-MS/MS methods (Paul et al., 2010) (Paul et al., 2008), which differ from one another only in the amount of sample used (respectively 20 and 10 mg), the derivatizing agent was BSTFA, and the monitored transitions for quantitation were 261 → 134 for EtG and 266 → 143 for EtG-d5, while no qualifier transitions were reported to unambiguously identify the targeted analyte. While this choice aimed to obtain the highest signal and the lowest limit of detection (LOD), in our testing with BSTFA, these transitions yielded unsatisfactory S/N ratios resulting from a high chromatogram baseline and led to major limitations in the quantification of low concentration samples.

A different derivatizing agent, namely MSTFA, was employed in our definitive method. The full-scan EI spectrum of the resulting EtG-TMS tetra-substituted derivative shows an abundant fragment ion at m/z 405 generated by the molecular ion at m/z 510 from the consecutive losses of a methyl radical [CH_3_, arising from a (CH_3_)_3_Si- group] and a (CH_3_)_3_SiOH molecule, which represents a common observation for glucuronide derivatives (Lai and Fiehn, 2016). Similarly, the isotopically-labeled EtG-D5-TMS derivative shows a fragment ion at m/z 410 arising from the m/z 515 molecular ion. These two fragment ions were used as precursor ions in the acquisition of several product ion spectra corresponding to different values of collision cell voltage. From the m/z 405 precursor, a characteristic fragment ion at m/z 359 was identified, corresponding to the loss of an ethanol molecule (m.w. = 46). The same fragment at m/z 359 is present in both the EtG-TMS (from m/z 405) and EtG-D5-TMS (from m/z 410) product ion spectra since all deuterium atoms are located on the ethanol substrate.

**FIGURE 1:**
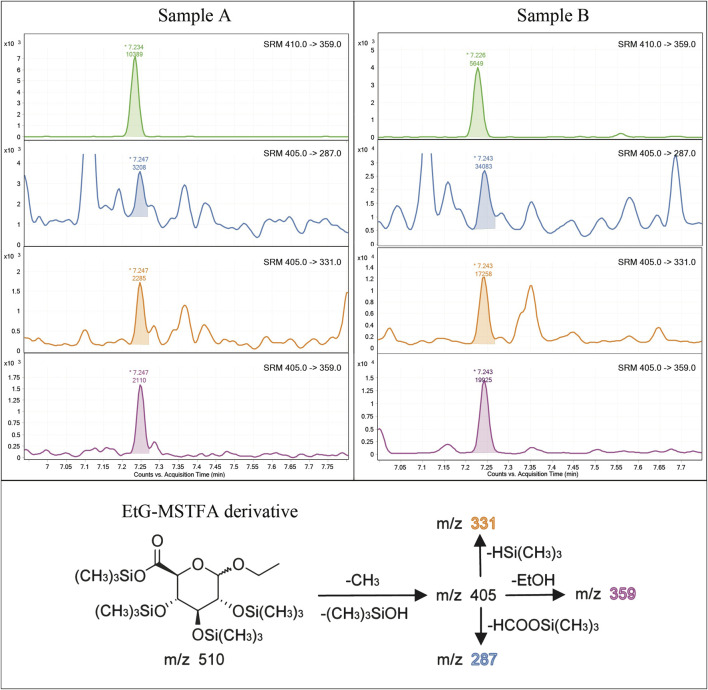
Example of chromatograms acquired in the MRM mode on a 6 pg/mg (LOQ) spiked (A) and a real hair sample of a strong drinker subject (B) with an estimated EtG concentration of 127 pg/mg. For each sample are reported, from the top to bottom, SRM transition of IS (EtG-D5), two qualifier SRMs, and the qualifier SRM. The fragmentation mechanism of the derivatized EtG and the related ion fragments of the selected SRM transitions are also shown in the lower part of the figure.

After this optimization, the selected reaction monitoring (SRM) program was set, using m/z 405 → 359 and m/z 410 → 359—both at 10 V collision energy—for EtG-TMS and EtG-D5-TMS quantitation, respectively. The transitions m/z 405 → 331 and m/z 405 → 287—again at 10 V collision energy—were used for EtG-TMS confirmation (Figure 1).

### Effect of Different Treatment and Extraction Procedures

One of the purposes of our study was to optimize the sample treatment procedure in order to guarantee the best analytical performance. Therefore, we evaluated the effects of different washing procedures, hair grinding, and incubation on EtG extraction. In particular, possible EtG loss during hair wash and the lack of EtG recovery from the matrix due to particle hair size or incubation conditions were investigated. This optimization was conducted using a purpose-oriented experimental design. The full factorial DoE results, conducted on hair samples from three different donors with different EtG levels, are summarized in Table 3. Most of these results are in agreement with the conclusions concerning the effects of the single variables reported in the literature, but the DoE design is expected to take into account combined multifactorial effects as well, as we experimentally verified.

**TABLE 3:** Factorial DoE results applied to the hair of three subjects.

Subject 1—Heavy Drinker, Grizzled ≈ 10 cm hairs						
Pretreatment conditions/Factor	(a) Washing procedures	(b) Incubation	(c) Shredding	Results (pg/mg)	Factor Value	Significant (t-test, *n* = 6 *α* = 95%) **>(19,71)**
0	−	−	−	81.8	−	−
a	+	−	−	74.9	3.55	No
b	−	+	−	130.6	34.95	Yes
c	−	−	+	180.8	66.95	Yes
ab	+	+	-	168.0	6.5	No
ac	+	−	+	181.8	−11.7	No
bc	−	+	+	188.9	−36	Yes
abc	+	+	+	171.6	−	−

**Subject 2—Heavy Drinker, Brown ≈ 5 cm hairs**						
Pretreatment conditions/Factor	(a) Washing procedures	(b) Incubation	(c) Shredding	Results (pg/mg)	Factor value	Significant (t-test, *n* = 6 *α* = 95%) **>(10,82]**
0	−	−	−	33.3	−	−
a	+	−	−	46.1	−1.25	No
b	−	+	−	70.2	13.47	Yes
c	−	−	+	89.7	24.59	Yes
ab	+	+	−	71.2	0.23	No
ac	+	−	+	73.9	−8.11	No
bc	−	+	+	79.2	−17.52	Yes
abc	+	+	+	76.3	−	−

**Subject 3—moderate drinker, black ≈2 cm hairs**						
Pretreatment conditions/Factor	(a) Washing procedures	(b) Incubation	(c) Shredding	Results (pg/mg)	Factor value	Significant (t-test, *n* = 6 *α* = 95%) **>[6,03]**
0	−	−	−	15.4	−	−
a	+	−	−	17.1	−1.64	No
b	−	+	-	25.7	3.19	No
c	−	−	+	33.0	9.76	Yes
ab	+	+	−	25.5	−1.66	No
ac	+	−	+	31.3	−2.39	No
bc	−	+	+	32.4	−6.16	Yes
abc	+	+	+	26.0	−	−

The factor value was calculated for each set of parameters as described by Douglas C. Montgomery in Design and Analysis of Experiments (2013) and compared with the significance result obtained with the t-test. Each test is named with the letter relative to the variable set high (+) (e.g., in the “ab” test, the sample was washed with 5 ml CH_2_Cl_2_ followed by 2 × 5 ml CH_3_OH, snipped with scissors, and incubated overnight at 60°C). (0) represents the three pretreatment steps with a low treatment level. Similarly, each factor value is named with the letter corresponding to the variable subjected to a verification of significance (or a correlation between two factors: ab, bc, or ac). The explanation for low and high levels and the (+) and (−) signs is shown in Table 2.

The effect of the washing procedures did not show any significant decrease or increase in EtG recovered from hair, as is evident by comparing the (0) vs. (a), (b) vs. (ab), and (bc) vs. (abc) results. Actually, the use of a single and mild washing procedure does not result in significantly higher EtG concentrations with respect to a quadruple washing protocol, ruling out both the occurrence of external EtG contamination and washout effects on EtG incorporated in the inner part of hair.

As regards the shredding (variable c) and incubation conditions (variable b), the DoE experiments showed a clear effect of ball-mill pulverization with respect to manual cutting under the same incubation conditions, as evident by comparing (c) vs. (0) and (ac) vs. (a), and (bc) vs. (b) results. Moreover, a strong correlation was found between variables b and c (factor bc), which showed a statistically significant effect in all tests. On the other hand, the effect of the incubation conditions is evident in (b) vs. (0) and (ab) vs. (a) results, but no improvement is observed between (bc) and (c) conditions. This means that in pulverized hair samples, the treatment with ultra-sonication for 2 h is sufficient to extract all or most of EtG, since no increase is observed with overnight incubation. In contrast, the hand-cut samples show a 40% lower extractive yield when 2 hours of extraction under ultra-sonication is carried out instead of overnight incubation. Overnight incubation of cut hair (b and ab) apparently provides an extractive yield 8–28% lower than 2-h sonication of pulverized hair (c and ac). Thus, pulverization with a ball mill is recommended whenever exhaustive extraction is required.

**FIGURE 2:**
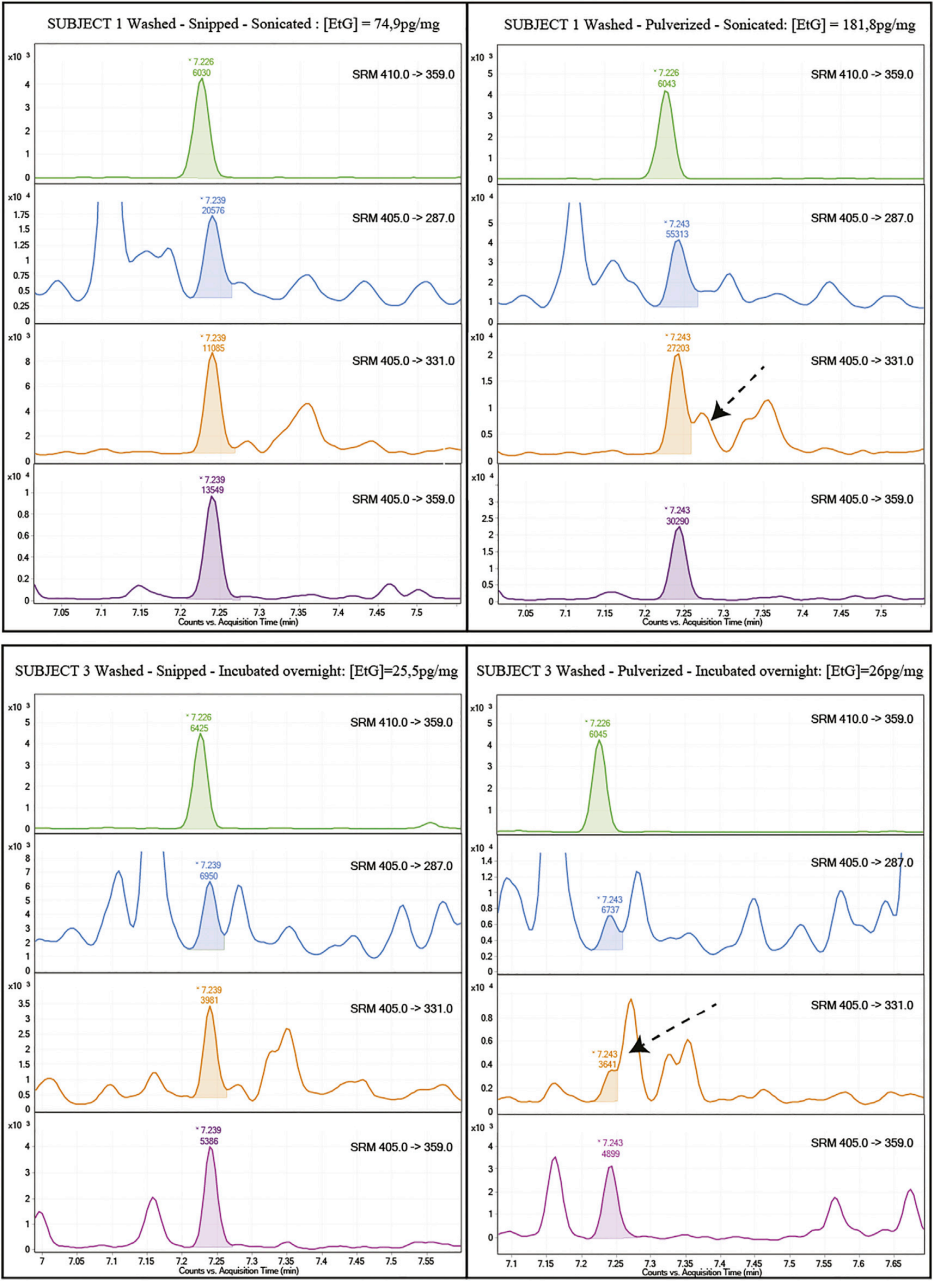
Example of chromatograms obtained from the analysis of the same hair sample (top: subject one tests “a” vs. “ac”; bottom: subject three tests “ab” vs. “abc”) pulverized or shredded. The arrow highlights the interference on the qualifying MRM 405→331 on pulverized samples regardless of the washing or incubation procedures (orange lines). Other MRMs were also reported for all tests.

Examination of the chromatograms evidences a strong interference on the m/z 405→331 qualifier transition for all the pulverized samples, regardless of the washing procedure or incubation (Figure 2). This interference was probably caused by the extraction of endogenous components following the breakage of the hair fibers by pulverization. However, it is recommended that SRM methods implemented in MS/MS instrumentation include at least one (not two) qualifying transition, which in the present case is fulfilled by the m/z 405 → 287 transition. Interference from endogenous hair components has rarely been observed in LC-MS-based methods.

In order to improve the extraction efficiency from cut hair while assuring the chromatographic quality, the same three hair samples were subjected to a further test in which manual cutting and overnight incubation were followed by 2 h of sonication at 50°C. This condition of double extraction did not show any recovery enhancement with respect to the samples only incubated overnight, demonstrating that further sonication after overnight incubation is not significant.

In conclusion, manual cutting and overnight incubation guarantee good chromatographic quality on all MS/MS transitions and complete compliance with both identification parameters (ratio of the two qualifier transitions to the quantifying transition) but produce partially incomplete EtG extraction.

### Validation of the Analytical Method

**FIGURE 3:**
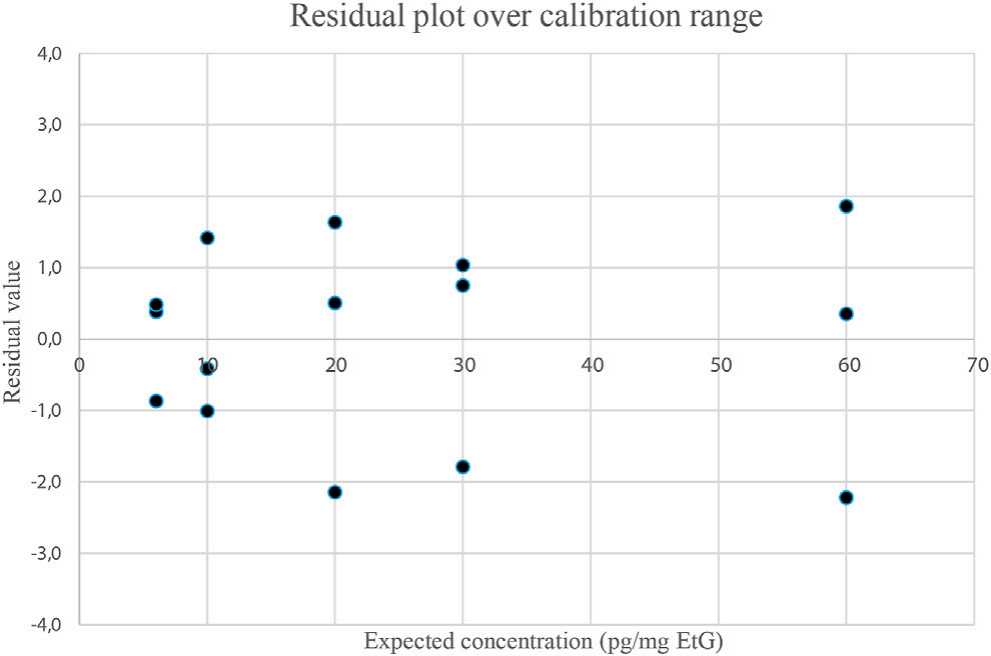
Residue plot of calibration points (3 replicates per level) calculated by subtracting from the expected value the concentration obtained with the curve. Residue values were then plotted against the expected concentration.

Previous methods based on GC-EI-MS/MS (Paul et al., 2008; Paul et al., 2010) used BSTFA for EtG derivatization and reached a LOQ of 10 pg/mg and a LOD of 5 pg/mg, at best. The present analytical method proved capable of detecting EtG in hair samples across a wide range of concentrations. Examples of chromatograms from a blank hair sample spiked at 6 pg/mg (LOQ) and a real positive hair sample at a high concentration (127 pg/mg) are shown in Figure 1. The calibration range extends from 6 to 60 pg/mg, with an estimated LOD of 4 pg/mg, which is experimentally confirmed. The calibration range proved linear both by visual inspection of the residue values (Figure 3) and by lack-of-fit and Mandel tests (*p* < 0.05) and covers the expected EtG concentrations for sporadic, social, and excessive drinkers, even if it cannot be legally used to support a declared abstinence, which would require a LOQ equal to or lower than 5 pg/mg (Society of Hair Testing, 2016). No EtG above the LOD was found in the blank controls. Hair EtG concentrations higher than 60 pg/mg were determined both by dilution of the final extract and using a different calibration curve, extending from 60 pg/mg to 300 pg/mg, with equivalent results.

Excellent intra- and inter-assay precision of the method, expressed as relative standard deviation, was proved by values lower than 15% over the calibration range (Table 4). Also the trueness, expressed as bias, proved to be satisfactory (Table 4), showing figures consistently lower than 10% and indirectly confirming the quality of the calibration curve. Intra-assay precision and trueness were also determined at the LOQ level (6 pg/mg) by six repeated experiments on spiked blank samples: a 5.66 pg/mg average value (bias 6%) was obtained, with CV% = 11%.

**TABLE 4:** Validation results for EtG in human hair samples in GC-MS/MS.

Expected concentration (pg/mg)—spiked blank matrix samples	Intra-day replicates (*n* = 3)			Inter-day replicates (*n* = 9)		
	Measured concentration (mean ± SD)	RSD (%)	Bias (%)	Measured concentration (mean ± SD)	RSD (%)	Bias (%)
20	19.9 ± 3.1	15	0.5	18.4 ± 2.6	14	8.2
30	31.8 ± 2.2	10	3.2	27.8 ± 2.7	10	7.3
60	65.9 ± 5.2	9	5.9	56.6 ± 4.3	8	5.6

### Correlation Between Ethyl Glucuronide in Hairs and Drinking Habits

**FIGURE 4:**
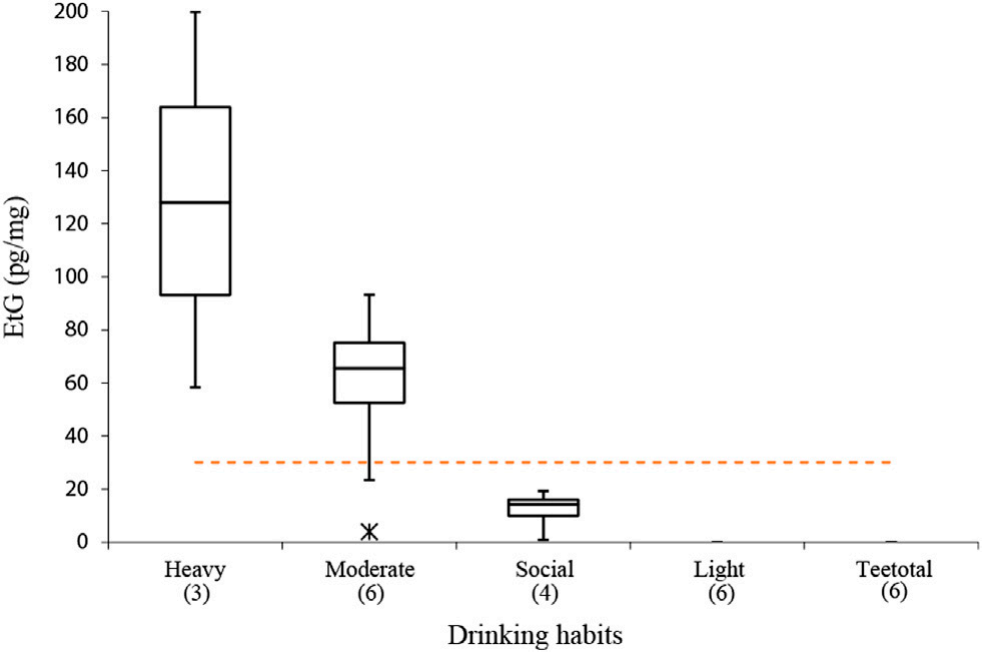
Box and whisker charts of EtG detected in the subjects compared to the subdivision into classes of alcoholic habits obtained on the basis of the questionnaire completed by each donor subject. Dashed line represents the SoHT cut-off value of 30 pg/mg and asterisk represents the outlier values for CB2 and CB3. Numbers in brackets represent the number of subjects for each group.

The quantitative results of the analysis conducted on real hair samples to provide a preliminary evaluation of the correlation between declared drinking habits and EtG levels are described in Table 5 and Figure 4. Even considering that the population sample was small, it is still possible to identify a substantial coherence between the average amount of alcohol affirmatively consumed by the donors and the level of EtG detected in the hair, with the exception of the subjects identified with CB2 and CB3. Even if a consistent variability in EtG concentration among the different subjects belonging to the same category is evident, it is noteworthy that the three sampled “heavy drinkers” exhibited EtG concentrations in the range 58–200 pg/mg, largely in excess with respect to the 30 pg/mg cut-off suggested by SoHT for chronic excessive drinkers. On the other hand, for all twelve teetotalers and light drinkers’ hair, EtG values below the LOD or LOQ were found, as expected. In particular, true teetotalers proved to have hair EtG levels around or below 1 pg/mg (Pirro et al., 2013); therefore, it is not unlikely that a large part of light drinkers’ testing turns out negative (i.e., below LOD = 4 pg/mg) due to the limited sensitivity of the present method. The class of “moderate drinkers” showed the largest result variability, which is frequently observed in relation to the difficulty of making an objective self-evaluation of the average alcohol consumption. In terms of results interpretation, it is noteworthy to consider the effect of cosmetic treatments.^1^ In our study, five out of six subjects who declared previous cosmetic treatments resulted negative (<LOD). Only one case (CB3) also reported moderate use of alcohol, while the remaining five volunteers stated light consumption. Therefore, it is likely that, in our cases, the cosmetic treatments did not affect the final EtG concentration.

**TABLE 5:** General information of the donors and data related to the hair collected. The last two columns relate to the classification of alcoholic habits derived from the questionnaire completed by each subject and the quantity of EtG found in the hair.

Id	Age	Gender	Sample hair length (cm)	Color	Cosmetic treatment	Drinking behavior classification	EtG in hair (pg/mg)
CB1	54	M	6	Grizzled	No	Social	14.9
CB2	58	M	5	Grizzled	No	Moderate	<LOD
CB3	37	F	6	Brown	Yes	Moderate	<LOD
CB4	46	F	6	Brown	Yes	Light	<LOD
CB5	55	F	6	Brown	Yes	Moderate	23.4
CB6	55	M	6	Grizzled	No	Heavy	127.9
CB7	56	M	5	Grizzled	No	Heavy	199.8
CB8	36	M	5	Brown	No	Moderate	93.2
CB10	45	M	6	Grizzled	No	Heavy	58.3
CB11	54	M	6	Grizzled	No	Light	<LOQ
A	14	F	4–5	Brown	No	Teetotal	<LOD
B	46	F	6	Black	Yes	Light	<LOD
C	44	F	6	Black	No	Social	19.3
D	49	M	4–5	Dark brown	No	Moderate	62.1
E	18	M	3	Brown	No	Social	<LOD
1 FRA	57	F	6	Brown	No	Teetotal	<LOD
1 FIL	17	M	6	Blond	Yes	Teetotal	<LOD
1 GIA	17	M	6	Brown	No	Teetotal	<LOD
F	13	M	2	Dark brown	No	Teetotal	<LOD
G	48	F	6	Dark brown	Yes	Light	<LOD
H	16	F	6	Brown	No	Teetotal	<LOD
I	52	M	3	Grizzled	No	Light	<LOD
AA	59	F	6	Grizzled	No	Light	<LOD
BB	59	M	3	Brown	No	Social	13.3
M	53	M	5	Grizzled	No	Moderate	69.1

## Conclusion

The continuously increasing relevance gained by EtG determination in hair for the assessment of chronic excessive alcohol drinking entails the availability of an array of assorted analytical methods matching the instrumentation accessible in the different toxicology laboratories throughout the world.

The GC-EI-MS/MS method successfully developed and validated in the study presently described exhibited analytical performances adequate to identify heavy and moderate drinkers and discriminate them from social and light drinkers and teetotalers. Upon careful optimization of the pre-analytical treatments applied to the hair matrix, the resulting analytical performances made it possible to obtain detection limits and quantitative accuracy comparable with those obtained with LC-MS/MS methods, granting the opportunity to carry out EtG analysis even in laboratories that lack this instrumentation. With respect to the GC-MS/MS methods that make use of electron capture NCI, electron impact ionization offers better signal stability and inter-day reproducibility.

In agreement with previous studies present in the literature, the recovery of EtG from authentic hair specimen proved highly dependent on the extraction conditions. Hair aliquots from the same subjects treated with different crushing end extracting protocols yielded significantly dissimilar quantitative results, possibly leading to different classification of alcohol consumption. Therefore, a standardization of the preliminary hair sample treatments should be established in order to reduce intra- and inter-laboratory variability and improve the quality and acceptance of EtG analysis. In particular, the parameter that proved to have the highest influence on the analytical results from the tested hair samples was the hair crumbling technique, in agreement with the international consensus from the Society of Hair Testing. The procedure of hair pulverization in a ball mill allowed us to reduce the time needed for the subsequent extraction step, without significantly affecting the EtG extraction recovery.

## Data Availability

The raw data supporting the conclusion of this article will be made available by the authors, without undue reservation.

## Footnotes

1 Society of Hair Testing (2019): https://soht.org/images/pdf/Revision_2019_Alcoholmarkers.pdf (lass access 13th February 2022).
